# Perspectives on Creating a Chronic Pain Support Line in Portugal: Results of a Focus Group Study among Patients and Healthcare Professionals

**DOI:** 10.3390/jcm13175207

**Published:** 2024-09-02

**Authors:** Mariana Cruz, Maria Inês Durães, Patrícia Azevedo, Célia Carvalhal, Simão Pinho, Rute Sampaio

**Affiliations:** 1 Department of Biomedicine, Faculty of Medicine of University of Porto, Portugal Alameda Prof. Hernâni Monteiro, 4200-319 Porto, Portugal; 2 Centro Hospitalar Universitário de Coimbra, Praceta Prof. Mota Pinto, 3004-561 Coimbra, Portugal; 3 Administração Regional de Saúde do Norte, R. de Santa Catarina 1288, 4000-477 Porto, Portugal; 4 Centro Hospitalar Tondela Viseu, Av. Rei Dom Duarte, 3504-509 Viseu, Portugal; 5 CINTESIS@RISE, Rua Dr. Plácido da Costa, 4200-450 Porto, Portugal

**Keywords:** chronic pain, helpline, focus group, qualitative research

## Abstract

**Background:** Chronic pain (CP) patients frequently feel misunderstood and experience a lack of support. This led to the creation of support telephone lines in some countries. However, there is no scientific data grounding their development or evaluating their performance. Almost 37% of the Portuguese adult population suffers from CP, with great costs for patients and the healthcare system. **Methods**: To determine the viability of a support line for CP in Portugal, a qualitative study was designed, and online focus group meetings, with patients and healthcare professionals, were conducted. Their perspectives, beliefs, and expectations were evaluated and described. **Results**: This study revealed that a CP support line is a feasible project from the participants’ perspective if its interventions are limited to active listening, emotional support, and tailored suggestions. **Conclusions**: It has the potential to generate a positive impact on healthcare services, while also contributing to greater equity of access to support.

## 1. Introduction

The International Association for the Study of Pain (IASP) defines pain as “an unpleasant sensory and emotional experience associated with, or resembling that associated with, actual or potential tissue damage”, identifying it as a leading cause of suffering and disability in the world [1,2,3]. Chronic pain (CP) was recognized by the European Federation of Pain (EFIC) as a pathology in 2001, whether isolated or accompanied by other disorders [4]. It is defined as pain that persists or recurs for longer than 3 months, associated with significant emotional distress or functional disability [2]. CP can be classified as primary CP (without an obvious cause) or secondary CP (as a symptom of an underlying pathology, including neuropathy, cancer, post-surgical, post-traumatic, orofacial, visceral, and musculoskeletal pain) [2].

The prevalence of CP is as high as 20% in the European adult population, while similar percentages can be found in the USA [5,6]. A Portuguese population-based study revealed that almost 37% of adults suffer from CP, making it a major public health issue [7]. The consequences of CP are vast, as they are both personal, economical, and societal, influencing absenteeism, social benefits, and early retirement costs. The annual cost of CP in Portugal amounts to EUR 5635.26 million; however, only 1% of Portuguese pain patients have access to specialized healthcare [8]. It has also been shown that, while patient access to care is somewhat restricted, when someone uses health services due to their pain, there is a propensity for overuse with no clinical gain [8]. Such findings make it pertinent to propose new intervention programs by bringing patients and healthcare professionals closer, empowering those who suffer, and maximizing the effective use of the available resources [7,8].

The creation of a CP support line (CPSL) can be one such program, making it possible to avoid some of the unjustified use of healthcare services (by providing patients with pain-preventing tools and self-management) and tackling major issues related to CP management, such as medication adherence (i.e., intaking the correct dosage of the right medicine in a timely manner and for the prescribed duration). Some pain support lines are functioning already, in countries such as Ireland, Canada, England, and Australia. Most were created by private organizations and operated by volunteers [9,10,11,12,13]. Even though this type of program seems to have a positive impact on patients with CP, with testimonies reinforcing the importance of human presence for those suffering, there is a lack of objective data on their effectiveness [9,14,15,16].

Using focus group methodology to gather an in-depth understanding of the perceptions and beliefs of patients with CP and healthcare professionals working in the field, we believe it to be possible to provide a truly objective ground for creating such a service. The focus group is characterized by a close interaction between the moderator and the group members and interactions between the participants themselves [17,18,19,20]. This methodology makes it possible to better understand the participants’ perceptions on the topic under discussion and is particularly useful in the context of healthcare research, as most health-related conditions depend on the social environment and context [18,19]. Focus groups have been widely used to gather knowledge regarding patients’ experiences with health services and healthcare providers’ attitudes and needs [19,21].

This study aimed to describe the perspectives of both patients with CP and healthcare professionals regarding the creation of a CPSL in Portugal. To our knowledge, this is the first time the feasibility of a CP support line has been studied and structured within a scientific approach. Focus groups followed a previously published protocol [22], allowing for the development of a support line based on the reality of those living with the disease. Gathered data will also provide insight into the potential impact of a CPSL.

## 2. Materials and Methods

The present study is part of a larger project titled “Creating a pain support line in Portugal: feasibility, development and impact” and reports the results of focus groups carried out with patients with CP and healthcare professionals working in the field. The main objective was to evaluate the feasibility of creating such a support line, according to the participants’ perspectives, beliefs, and expectations.

### 2.1. Study Design and Setting

To assess patients with CP and healthcare professionals’ needs and expectations, an exploratory and phenomenological qualitative design based on focus group interviews was used, following the COREQ (Consolidated Criteria for Reporting Qualitative Research) guidelines [23,24]. An approach based on focus groups was considered to be the best option, as it allows researchers to obtain a varied array of perspectives, as close to everyday reality as possible, while providing participants with a safe and familiar environment to express their opinions and describe their experiences [19,21]. Due to COVID-19 pandemic restraints and post-pandemic time constraints, focus groups were arranged in an online setting, using the Zoom^©^ videoconferencing platform. According to some authors, it is recommended that online focus groups do not exceed five to six participants because a fluid discussion is difficult to achieve [25,26]. The focus group dynamic and videoconference characteristics and requirements were presented before the meetings to both patients and healthcare professionals who volunteered to participate. All participants were informed that meetings were being video recorded for later transcription and analysis, under the Portuguese data protection laws.

Qualitative data were obtained and analyzed with the NVivo^©^ software, version 12, and evidence was collected regarding the feasibility of developmental aspects of a support line.

### 2.2. Recruitment and Sampling

Patients registered in the *Força3P* pain association were invited to participate by e-mail, with the help of the association’s representative. Following the same rationale, healthcare professionals working in public health units in Portugal were also invited by e-mail. The focus groups took place at a time conveniently arranged for most of the participants, who had previously communicated available timeframes for scheduling. All participants received a presentation explaining the study context and objectives and gave informed written consent after the presentation and prior to the interviews. Participants were selected through convenience sampling.

### 2.3. Participants

The inclusion criteria of participants consisted of them being 18 years old or older, having the ability to understand and communicate in Portuguese, being able to use technologies and to log into a Zoom^©^ call, and being a patient with CP (diagnosed at least two years ago) or a healthcare professional working in Portuguese public healthcare units who attend to patients with CP.

#### 2.3.1. Healthcare Professionals

In total, twenty-one medical doctors and nurses were contacted. Twelve volunteered to participate, one refused, and the others did not respond. They were e-mailed a short presentation of the project and a date suitable for the majority was arranged. Written consent was obtained prior to the meeting. Two of the volunteers were unexpectedly called to emergency cases; therefore, meetings occurred with the participation of a total of nine doctors and one nurse, within two meetings (*n* = 4 and *n* = 6). Within the specifications of qualitative research, we consider theme saturation to have been reached in these groups.

#### 2.3.2. Patients with CP

Regarding patients with CP, seven volunteered to participate, but only six (*n* = 6) were effectively present. No further patients were recruited as theme saturation was reached.

### 2.4. Data Collection Procedures

The focus group meetings were held on the Zoom^©^ platform (online videoconference) and led by two researchers. An entirely online approach was chosen to ensure safety and flexibility in the pandemic and post-pandemic era. The two researchers were present 30 min before the starting time to solve any technical issues. The facilitator was responsible for hosting and moderating the discussion, by asking questions from the discussion guide and ensuring adequate individual participation. The second moderator was responsible for recording major quotes, non-verbal interactions, and expressions, to add context to the recordings and aid the discussion of the transcripts later.

Questions related to the search topic were based on the literature regarding patients with CP and healthcare professionals’ experiences, as well as related to technological potential and applications in medicine and CP [16,27,28,29,30,31]. The question guide used in each focus group was discussed in previous work [22] and did not limit the scope of the discussion; it was merely used to stimulate dialogue further.

The interview duration was one hour and forty minutes in the focus group of patients with CP and an average of one hour and thirty minutes for healthcare professionals’ groups. All participants opted to display their real names, and all interviews were audio and video recorded. Everything was anonymized afterward. A total of three focus groups were carried out: one with patients and two with healthcare professionals. Data saturation was attained.

### 2.5. Analysis

The recordings were transcribed verbatim, anonymized, and codified; they were not returned to participants. Analysis was made according to the framework method [32,33]. Findings emerged directly from raw data, based on both inductive and deductive coding; each technique was applied independently for the patients’ and healthcare professionals’ focus groups in a systematic, sequential, and continuous way [34]. Data analysis with QRS NVivo^©^ versions 12 and 14 software enabled identifying and organizing codes, categories, and themes. Coding of the segments of the transcriptions was made quotation by quotation [35]. The segments of coded text were synthesized into categories, which were further grouped into major themes [36,37]. Thematic analysis was performed through an iterative and reflexive process, carried out by three of the researchers, independently of each other. Afterward, comparative analysis between focus groups was carried out to better understand how the perspectives of patients with CP and healthcare practitioners converged or diverged in specific topics. All data were discussed and interpreted by at least two researchers; any disagreements were discussed with a third researcher until a consensus was reached. Participants were not asked for feedback on the findings. All quotes presented were transcribed from Portuguese to English and double-checked.

### 2.6. Data Sharing

Data retrieved from focus groups were recorded, transcribed, and completely anonymized. It includes the complete verbatim transcriptions, which will be made available upon request. The study protocol has been published [22]. There are no frequencies of categorical variables to report, as our approach primarily dealt with establishing concepts rather than specific wordings; this is because participants used different wording to convey the same concept, feeling, and/or opinion, and hence the importance of not limiting the analysis to a frequency determination. As stated previously, two coding methods were used (inductive and deductive), by manual codification with NVivo^©^ software. There was no statistical analysis, the final data were analyzed through a comparative in-depth discussion by at least three researchers.

## 3. Results

In total, three focus group interviews were conducted. The majority of patients with CP were females (83%) and the same percentage were married. Fifty percent (50%) were unemployed and 33.3% had a university degree. Regarding the 10 healthcare professionals (*n* = 10): there was a nurse, an anesthesiologist, a stomatologist, a physical and rehabilitation medicine physician, and six family medicine physicians. The experience working with patients with CP ranged from 5 to 20 years.

After transcription, the data were coded, first inductively and then using the deductive method to confront the results and minimize bias. Four major themes emerged in both approaches and the three focus groups: (1) support available for patients, (2) perceived patients’ difficulties, (3) perceived patients’ necessities, and (4) creation of a chronic pain support line. The major theme “Perceived patients’ difficulties” was discussed to a lesser extent in the healthcare professionals’ groups. Descriptions can be found in Table 1.

As for the minor themes, some appeared only with deductive coding, reinforcing their less significant role. These themes are related mainly to two topics: the daily living of patients and the importance of treatment adherence on pain control. This last theme was only discussed in the healthcare groups but its relevance for pain management and its dependency on the doctor–patient relationship and proper communication was vehemently reinforced.

### 3.1. Theme 1: Support Available for Patients

Under Theme 1, four types of support were identified: Hospital pain units; Family physician (FP), which was much more valued by healthcare professionals than by patients; Emergency Services, which were solely mentioned by patients (even though with a negative outlook on their efficacy) but never mentioned by professionals; and Information available about CP, which was considered to be insufficient by patients while healthcare professionals regard it as adequate. All in all, perspectives slightly diverged between the two groups, and examples of the two can be found in Table 2.

### 3.2. Theme 2: Perceived Patients’ Difficulties

Five categories emerged under the perceived patients’ difficulties: Feeling misunderstood, which was identified as a major drawback by both patients and professionals; Difficulty in asking for help, admitted by almost all patients but unnoticed by healthcare professionals; the majority of patients mentioned Misjudgment of complaints and delay of diagnosis, which was also recognized by health professionals, who described patients with CP as frequently having undergone multiple medical assessments and treatments achieving no effective response nor adequate follow-up, which contributes to the feeling of discredit; Difficulty accepting the diagnosis, which is seen as a source of suffering for patients, but somewhat overlooked by professionals; and Gender bias, a common complaint among female patients, failed to be mentioned by healthcare professionals. Examples of these opinions are described in Table 2.

### 3.3. Theme 3: Perceived Patients’ Necessities

Patients and healthcare professionals discussed six major necessities: Emotional and psychological support was identified as a crucial need, mainly during pain exacerbations; Effective communication skills, such as active listening, empathy, and avoidance of a patronizing attitude were mentioned as essential qualities of healthcare professionals; Scientifically reliable information was also identified as an important need, with all three groups stating it should be provided by professionals and easily available for patients. Regarding this topic, healthcare professionals highlighted the need for adequate training and on-the-job opportunities to keep updating their knowledge; Advice and coping mechanisms on how to deal with pain and its exacerbations, as well as how to prevent them, were mentioned in all groups; and Limitless support, as all participants agreed that there is a need to have permanent access to support because pain exacerbations are impossible to anticipate. Both patients and professionals emphasized the Opportunity for sharing common experiences, as it seems to be a source of relief and understanding for patients. Excerpts of these topics are listed in Table 2.

### 3.4. Theme 4: Creation of a CPSL

With respect to the creation of a CPSL, five topics emerged: Support line functionality was a recurrent topic in which participants reinforced the importance of adequate skill training for those operating the line, such as having pharmacological knowledge and high-level communication skills. Professionals considered the possibility of creating an algorithm-based CPSL not to be feasible, as it would narrow the scope of the support line, which must also be very well defined. All three groups identified various Support line requirements: the permanent availability of support and the possibility of receiving video calls, and the option for patients to remain anonymous. Moreover, callers should have the opportunity to choose the sex of the operator and the advice given should not only be evidence-based but also tailored to the individual. Participants also said that a CPSL ought to be able to connect them to a CP Patient Association or provide them with tools to contact fellow CP patients (which reinforces the importance of sharing common experiences). Healthcare professionals also mentioned that professionals must have adequate training and that the line should act as a bidirectional way of exchanging information (e.g., sharing information with the patient’s family physician). As for What to avoid in a support line, patients and professionals clearly stated that a CPSL must never act as a replacement for an appointment with a doctor: diagnosing illnesses is unacceptable and the prescription of medications must never occur. Another reiterated idea was that patients’ complaints must never be dismissed. Foreseeable difficulties included the sizeable need of staff, which must be adequately trained and possess the necessary knowledge, the guarantee of availability without imposing limitations in call duration, and funding. Professionals also mentioned the fact that the only source of information during a call would be the patient, which would necessarily add subjectivity to the interaction and potentially impair one’s ability to provide adequate advice. Regarding Support line feasibility, patients stated that creating a support line is not only feasible but also desirable, to create equity among all patients. Healthcare professionals maintained the idea that a CPSL would be useful and could potentially contribute to lessening their burden. Examples can be found in Table 2.

Besides the main themes described so far, two other minor themes emerged. Among professionals, Healthcare professionals’ current difficulties were discussed: the participants mentioned having too many patients on their lists, too much administrative workload, and the fact that they often feel overburdened, particularly from an emotional perspective; when trying to help their patients, family doctors feel that they frequently give too much of themselves.

*“Because it is often the doctor acting as a therapeutic tool (…),I present myself full of energy, full of positivism, full of optimism, full of ideas, that I will be able to change the patient’s life (…). At the end of the day, I am a wreck!”*—Professional 2

*“I often feel empty… Completely consumed.”*—Professional 1

Healthcare professionals also mentioned difficulties in accessing patients’ information regarding their chronic pain, suggesting that an integrated data platform should be created and shared by all potential health services so that it would be possible to know the patient history of assessments and treatments. On the same rationale, healthcare professionals pointed out the absence of protocols or guidelines to support clinical practice regarding CP, as well as a lack of opportunities to keep on learning and improve their skillset in relation to the field.

*“(…) It is true, the lack of health education, not only among patients but also among us, professionals, makes it all the more difficult.”*—Professional 3

Among patients, the *Use of information technologies to support patients with CP* was discussed, relating to the idea of increasing healthcare access equality. Patients considered the use of information technologies to be an inexpensive widespread means to deliver support to more remote areas of the country.

*“(…) this line could be a way of having more equality, as it can be accessible anywhere in the country.”*—Patient 5

## 4. Discussion

This study describes the perspectives, needs, and difficulties that both patients with chronic pain and healthcare professionals experience on a daily basis. It also shows that creating a chronic pain support line for patients is considered valuable, as long as such a line operates under well-defined guidelines.

When coding focus group data, four major themes emerged. There was no difference in major themes when comparing patients’ and healthcare professionals’ focus groups. The fact that coding was made both inductively and deductively and that inductive coding was applied first strengthens the validity of collected data and increases the reliability of the findings, as bias was significantly reduced.

By analyzing the first main theme, Support available for patients, one can find interesting distinctions between patients’ and professionals’ perspectives of CP support. Firstly, both groups agreed that Hospital Pain Units are the best available support system for patients with CP. This is somewhat expected, as these units have teams specialized in dealing with CP; nevertheless, healthcare professionals highlighted that these units have very long waiting lists. The second most mentioned support system was family physicians. Here, opinions start to differ: healthcare professionals assume that, besides being responsible for most Portuguese patients with CP, FPs are mostly successful in managing their pain. This notion is corroborated by a previous study, which stated that 85% of Portuguese patients with CP are managed by FPs [38]. However, patients with CP have less trust in FPs’ ability to manage their pain, stating that they do not have the necessary knowledge about CP. Patients’ perspective is more in line with what has been demonstrated in previous studies [39,40]. Their lack of trust in their FPs leads them to resort to emergency services when experiencing pain exacerbations, even though they complain that these too are unsatisfactory support services. This is unsurprising since emergency services are designed to manage acute and at times life-threatening situations and CP patients’ needs are very different, so much so that healthcare professionals did not even consider emergency services to be a type of support for CP management. These findings show that patients often have misconceptions regarding the role of emergency services, which leads to their overuse, as the previous literature has described [16,38]. Another relevant inconsistency between patients’ and professionals’ perspectives concerns available information about CP and its management tools: patients refer to it as insufficient and difficult to access, whereas healthcare professionals believe the resources provided are enough. This may indicate an important gap in communication.

The second main theme discussed, Perceived patients’ difficulties, understandably had a greater emotional significance in the patient group. This is aligned with the Self-Regulation Model of Leventhal: when confronted with illness, people tend to process the information emotionally and cognitively to give it meaning and then cope with it [41]. As everyone has a unique illness representation, it is not surprising that a lack of understanding is perceived as a major issue, with patients revealing that they deal with other specific struggles in their everyday life that professionals failed to mention. A frequently discussed problem was the inhibition patients show in asking for help. This is a complex issue that may be partially explained by cultural beliefs regarding pain [9,39,40]. Misjudgment and dismissal of complaints, with consequent diagnosis delays, was another obstacle identified. This seems to be more frequent among women, who highlighted that male doctors are more prone to interpret their symptoms as secondary to psychiatric pathology [39,40]. This finding seems to suggest that there is a gender bias in CP management. Although this bias is not new, further studies are needed to clarify the motives behind these perceptions [40]. For different reasons, men also mentioned that it would be important to them to be able to choose the gender of the person they interact with. In their case, the main reason for this is that they feel there are sensitive themes they are not comfortable discussing with someone of the opposite sex, such as sexual dysfunction secondary to chronic pain. Finally, patients are also assumed to experience some reluctance in accepting their diagnosis once it is made. This may seem like a somewhat peculiar and even paradoxical notion at first, given the fact that the same patients complained about the excessive time to diagnosis; however, when one takes into account the fact that CP is a serious, often permanent, and difficult to manage diagnosis, it is easier to understand their reluctance.

Regarding the third major theme, Perceived patients’ necessities, there was greater unanimity. Both patients and healthcare professionals identified emotional and psychological support as the main necessity for those who suffer from CP. This seems to be linked to the misunderstanding of patients’ feelings, as well as to the burden of the disease itself [39,40,42]. Similarly, both groups mentioned the need for reliable and scientific information, which highlights the importance of adequate counseling and coping mechanisms: these can greatly help patients self-manage, besides aiding them in preventing their pain from becoming worse [16,39,43]. Patients also mentioned the importance of having support with no time limitations and good communication skills, ideas that have been widely discussed in previous studies [43,44]. Patients and healthcare professionals also noted the importance of being able to be in contact with people who have experienced similar situations and hardship.

Lastly, regarding the fourth main theme, Creation of a CPSL, there was also a significant alignment of ideas. Both groups agreed that those operating a CPSL must have proper training and experience in CP in particular [3]. Likewise, pharmacological knowledge is essential to help avoid mistakes and answer frequent questions. The possibility of creating contact points between patients and their peers or CP patients’ associations was also mentioned as an interesting service for a CPSL to provide; the importance of this approach has been discussed in previous studies [9,42,43]. Choosing the gender of the operator and making video calls were also mentioned as very significant positives from patients’ perspectives. The importance of video calls has become particularly relevant in recent years [45]. As for what to avoid, it was clear that the line must not make therapeutic interventions and must never act as a substitute for the doctor or nurse. Healthcare professionals added that algorithms are not reasonable in a support line that aims to support the experience of the disease and not the disease itself, as they would render the approach mechanical and almost exclusively directed at medical decisions, something that should be avoided, given the aim of such a project. Its purpose should be to complement already available support systems, with the potential to relieve the burden of the Pain Units and promote equality in CP support access. Both patients and professionals agreed on the feasibility of a CPSL. Professionals considered it a good system to relieve their work overload by making emotional and psychological support easily accessible to all patients. This idea has emerged in a previous study [16].

Despite the volume of data collected, participants rarely discussed medication adherence. It was only addressed by professionals after the facilitator mentioned it. Although this is an important factor in CP mismanagement, patients do not regard treatment adherence as relevant; as for healthcare professionals, they considered the assessment of medication adherence to be of major significance and openly assumed its dependency on the quality of the communication established with the patient, as described in previous studies [8].

This study has some limitations. The most obvious and major limitation was the sampling method; participants’ characteristics may be very similar regardless of their number, and thus the sample may not comprehensively represent the population being studied [34,46]. In this study, there were some societal groups that were underrepresented in the patients’ group: people from rural areas, males, and people with less than a secondary education. Nonetheless, the group included elements ranging from very talkative to very quiet, as well as people with diverging opinions on several topics, which enhances sample representativity. Regarding the study’s methodology, some aspects should be noted. Ideally, focus group meetings would have been conducted in person. This would allow us to better identify alternative sources of information such as participants’ body language and posture. However, it was necessary to opt for an entirely online approach to guarantee participants’ safety during the COVID-19 pandemic and more flexibility to enhance participation during post-pandemic restrictions.

The dropout rate was 14.3% for patients with CP, which is a relatively low number, compared to previous studies [47]. The higher participation rate might be explained by the logistical characteristics of online focus groups: they are easier to attend to and scheduling conflicts are less of a restriction [47]. Healthcare professionals’ numbers are compliant with the numbers reported in previous focus group studies, which accounted for a 52% dropout rate [47]. These findings confirm that a complete online approach to focus group methodology is valid and has the potential to enable research projects that would otherwise be impossible to undertake.

Despite these limitations, our findings provide insight into how receptive stakeholders are to the creation of a CPSL and how comprehensively it may respond to their needs.

To the authors’ knowledge, this is the first study supporting the development of a CPSL from a scientific standpoint; the project has the potential to have a positive impact on access equality and enhancement of quality of life for all patients with CP, while also contributing to more efficient use of healthcare services and healthcare professionals’ wellbeing.

## 5. Conclusions

Overall, the present study identified similar perceived needs between patients and professionals. However, two themes marked a distinct perception among the groups: gender bias regarding CP diagnosis and management (unidentified by healthcare professionals and not anticipated by researchers) and the need for more comprehensive health education among the Portuguese population, in order to prevent the overuse of emergency services associated with CP. Furthermore, bettering communication standards between patients and healthcare professionals also emerged as being of paramount importance. It also revealed that the creation of a CPSL is welcomed by both patients and healthcare professionals, as long as its development is accomplished in accordance with the identified needs and in close collaboration with all implied stakeholders. Both groups were also able to anticipate major difficulties in attaining a 24h support line, such us staff requirements and training needs, as well as the significance of the costs implicated in operating this type of support system.

## Figures and Tables

**Table 1:** Main themes’ descriptions.

Chronic Pain Patients	
Theme	Description
Support available for patients	Includes the existing support systems, their accessibility, and ideas on how to ameliorate them.
Perceived patients’ difficulties	Refers to the many difficulties patients with CP face, from the diagnostic to living with the disease for years.
Perceived patients’ necessities	Includes the main perceived needs to be addressed, identified by patients with CP.
Creation of a chronic pain support line	Includes opinions, characteristics that must be present, what must be avoided, and foreseeable difficulties from a patient perspective
Healthcare professionals	
Theme	Description
Support available for patients	Includes the existing support systems, their accessibility, and ideas on how to ameliorate them.
Perceived patients’ necessities	Includes the main perceived needs to be addressed, identified by healthcare professionals during their activity.
Creation of a chronic pain support line	Includes opinions, characteristics that must be present, what must be avoided, and foreseeable difficulties from a healthcare profession perspective.

**Table 2:** Perspectives of patients and healthcare professionals: excerpts.

Theme 1: Support Available for Patients
1.1 Hospital pain units “*I go to my pain doctor. It’s in the hospital, we have to call or if we show up we talk to the nurse. We always end up being attended to.”*—Patient 1 “*We also have a phone line for our patients (…) that turns out to be much more of a kind of psychological and emotional support*.”—Professional 2
1.2 Family physician “*That’s what’s important, is that at the Pain Unit they talk to us. The FP is very different; there is not much time for talking, as the doctor is overworked (…) giving the impression that he doesn’t even look at us.”*—Patient 5 “(…) *there is a minimal percentage of patients who are followed up in Pain Units. We dare say everyone else is followed by their FP.”*—Professional 1
1.3 Emergency services “*Going to the emergency services is a mistake because the professional who is there does not know how to assess our pain. If we have any other disease, he does not even waste time trying to understand what kind of pain the person has.”*—Patient 1
1.4 Information available about CP *“(…) because nobody talks to you about pain dedicated appointments, not in hospitals nor in primary health care centers.”*—Patient 5 *“I tell them to research this topic, consult this website (…). In our unit we have some leaflets that explain a little about pain and how to adapt to the pain situation, how the Pain Unit works, etc.”*—Professional 2
Theme 2: Perceived patients’ difficulties
2.1 Feeling misunderstood *“I used to go to the doctor, I complained of pain, but as I could not explain what I was feeling or verbalize the intensity and cause of the pain, it ended making me feel blocked (…).”*—Patient 3 *“I think people feel misunderstood. And since it’s not a visible thing, it is a problem that exists even within the family, is it not? The husband does not understand, the parents do not understand, nobody understands.”*—Professional 2
2.2 Difficulty in asking for help *“Experiencing what I was experiencing and what I was feeling was very complicated… asking for help? No way (…) what I have also seen is that people in this situation, especially in the early stages, are somewhat reluctant to seek healthcare.”*—Patient 3 *“I think people have difficulty…they think like “it’s pain, just bear it” and do not seek help (…).”*—Patient 1
2.3 Misjudgment of complaints and delay of diagnosis “*From the moment the person starts to feel it and looking for health professionals because she feels constant pain, until having a diagnosis, many years pass. First, they will investigate and discard all possibilities, until they decide we have CP because of an X or Y pathology. With all this, around 6 years go by*.”—Patient 3
2.4 Difficulty accepting the diagnosis *“I will give my example: before I was diagnosed and before I accepted CP I was angry, so for me a CP diagnosis was not seen with good eyes, despite actually having pain*.”—Patient 3
2.5 Gender bias *“If we are treated by male doctors, they have the idea that women are melodramatic, and very emotional, and the first thing they think of is always depressive syndrome. Sorry but this is true*.”—Patient 2
Theme 3: Perceived patients’ necessities
3.1 Emotional and psychological support *“In times of crisis and pain, we would just like to have someone we could talk to, to calm us down (…).”*—Patient 2 *“We also have a line for our patients (…) with strong psychological and emotional support, a lot of listening, giving expression to, allowing the expression of feelings”*—Professional 2
3.2 Effective communication skills *“Of course, listening is essential. The person on the other end must have an exceptional ability to listen, to respect the silences, have communication techniques, some psychotherapy tools.”*—Patient 7
3.3 Scientifically reliable information *“Dialogue is really important (…), and scientific information. Hence the importance of scales and pain assessment. (…) people must have scientific knowledge, I have no doubt about that.”*—Patient 2
3.4 Advice and coping mechanisms *“Sometimes we are so desperate and confused that the fact that a professional is asking basic and logical questions makes us open our minds and realize “I still haven’t done everything I could”. (…) I would like for the person to be an advisor and say: “Look, try this, try that, do this, apply hot water, apply cold water…”, a person who would guide us.”*—Patient 1 *“Just listening is not enough, if we do not implement strategies with our patient and try to get him to establish other focuses of attention, make him find other anchors in life besides his pain (…).”*—Professional 1
3.5 Limitless support *“I would have to know that there will be someone who knows how to listen and help during the entire process, without time limitations. (…) I want to feel that the person on the other end is holding my hand and will not let go until I am ok.”*—Patient 7
3.6 Opportunity for sharing common experiences *“I think that a group session/appointment would be a good option to consider. Chronic pain patients frequently state the need they have to feel understood, sharing experiences and stories would be a great option.”*—Professional 4
Theme 4: Creation of a CPSL
4.1 Support line functionality *“It is fundamental to have someone who deals with pain closely, because dealing with something is very different from learning about it and that makes all the difference (…). It would be good for those on the other end of the line to have knowledge.”*—Patient 3“ *We have to have very well-prepared people on the other end of the line, who understands what this concept of CP is, so as not to be immediately alarmed if the patient says he has 7 or 8 pain intensity.”*—Professional 1 *“Whoever is on the other end of the line has to have a very good understanding of analgesic schemes (…).”*—Professional 2“ *The issue with algorithms, for me, is that they will only lead to very clinical, very medical things, and we want listening, empathy, all not so mechanical (…). This [the scope of action of the line] must be extremely well-defined (…) we need to define it, so that there are no doubts on the part of the patient who calls (…). There must be clinical safety for both patients and professionals.”*—Professional 5
4.2 Support line requirements *“The support line should act throughout the entire process. (…) Whoever is on the line must know how to direct and adapt the advice given to the patient and his pathology.”*—Patient 3 *“It would be essential to be able to provide good quality information [to the patients].”*—Patient 2 *“(…) being able to ask for help anonymously and always respecting me as a person in pain.”*—Patient 7 *“Men may also want to be attended to by men… we have issues of daily life and sexuality.”*—Patient 6 (Male)
4.3 What to avoid in a support line “*The line could never give an authorization to do something, it should only give the correct information to the patient.”*—Patient 7 *“It makes sense if there is mostly active listening, coping mechanisms, not much more than that*.”—Professional 2
4.4 Foreseeable difficulties “*There needs to be someone, 24/7, available to answer calls. This part of the operationalization may be difficult to plan, it is necessary to mobilize a lot of people (…) and sponsorship to pay for the line. Oh, money, money!”*—Patient 2 *“Yes, it is important to have permanent possibility of contact, but in terms of resources it will be quite complicated.”*—Patient 7 *“But we have to beware, because superimposed on a well diagnosed CP can be an exacerbation of another disease, and that is a completely different story!”*—Professional 1
4.5 Support line feasibility “*Yes, I would use the line and try for the person [answering my call], in addition to the psychological support, to be my mentor (…).”*—Patient 1 *“For me the line makes sense like this [empathic listening and coping mechanisms] (…). I think that this line is not to solve problems, that is not the goal here.”*—Professional 2 *“I often hear the nurses complaining that we have patients who call there constantly, I do not say daily, but they do call two or three times a week.”*—Professional 2

## Data Availability

The data that support the findings of this study are available from the corresponding author, MC, upon reasonable request.
